# The rubber hand illusion in microgravity and water immersion

**DOI:** 10.1038/s41526-022-00198-4

**Published:** 2022-05-06

**Authors:** V. Bruno, P. Sarasso, C. Fossataro, I. Ronga, M. Neppi-Modona, F. Garbarini

**Affiliations:** 1 MANIBUS Lab, Psychology Department, University of Turin, Turin, Italy; 2 BIP Lab, Department of Psychology, University of Turin, Turin, Italy; 3 Neuroscience Institute of Turin (NIT), Turin, Italy

**Keywords:** Physiology, Neuroscience

## Abstract

Our body has evolved in terrestrial gravity and altered gravitational conditions may affect the sense of body ownership (SBO). By means of the rubber hand illusion (RHI), we investigated the SBO during water immersion and parabolic flights, where unconventional gravity is experienced. Our results show that unconventional gravity conditions remodulate the relative weights of visual, proprioceptive, and vestibular inputs favoring vision, thus inducing an increased RHI susceptibility.

We evolved on Earth, and our bodies have constantly been exposed to earthly gravity (1 g); hence, it has been suggested that the gravitational input significantly contributes to building a coherent sense of body ownership (SBO)^1^, i.e., the feeling that different body parts belong to one’s own body^2^. Reports from subjects exposed to altered gravity conditions show various illusory body perceptions yielding errors in body localization, body acceleration, and body configuration^3–5^. The present work aims to investigate whether and to what extent experimental procedures simulating altered gravity conditions, such as microgravity (a condition in which people or objects appear to be weightless) and water immersion (a condition used to simulate zero gravity), can affect SBO. To manipulate SBO, we employed the well-known rubber hand illusion^6^ (RHI) paradigm, where individuals watch a lifelike rubber hand being touched while their own hand, hidden from view, is touched synchronously. Our brain solves the multisensory conflict between the felt touch of one’s own hand and the vision of a touched rubber hand by incorporating the artificial hand, which is perceived as a part of the own body (i.e., embodied)^7–9^.

In order to modulate the gravitational input, the RHI was administered in two unconventional gravitational environments: (1) a parabolic flight (Parabolic flight experiment), in a campaign organized by the European Space Agency (ESA); and (2) immersion in water in a swimming pool (Swimming pool experiment). Both experimental contexts expose the nervous system to altered gravity conditions (see below), during which any difference in objective (a shift in the perceived position of the real hand) and subjective (the reported SBO over the rubber hand) measurements of the RHI may inform us of a gravity-dependent effect on the multisensory integration process subtending the SBO. In particular, we predict that the reduced gravitational pull will lead to an attenuated SBO, which, in turn, will increase the susceptibility to the RHI (i.e., embodiment of the fake hand). In the Parabolic flight experiment (sample: *n* = 5), the RHI was induced in short (12 s)^10^ stimulation sessions performed during both microgravity (0 g) and normal gravity (1 g) phases of each parabola (Fig. 1a). During parabolic flights, microgravity resembles 0 g and lasts about 20–25 s, whereas, in between parabolas, the aircraft flies in 1 g conditions (see Methods and Supplementary Information for the details about the acceleration). The feeling of weightlessness occurs when the airplane is in free fall only because in this condition, the aircraft’s speed and trajectory cancel out gravitational acceleration^11^. In the Swimming pool experiment (sample: *n* = 19), the RHI was performed during water immersion (Fig. 1c) and, as a baseline condition, out of the water on the ground (Fig. 1b). The water immersion condition was used for simulating zero gravity^12^ since it can influence body posture^13^, alter the habitual relationship between muscle activation and limb position^14^, and change the innervations of muscle spindles^15^. In both experiments, objective (proprioceptive drift) and subjective (embodiment questionnaire) RHI measures were collected after both synchronous (wherein the RHI generally occurs) and asynchronous (wherein the RHI generally does not occur, i.e., control condition) visuo-tactile stimulations of the real and the fake hands (see details in Methods and Fig. 1).

**Fig. 1:**
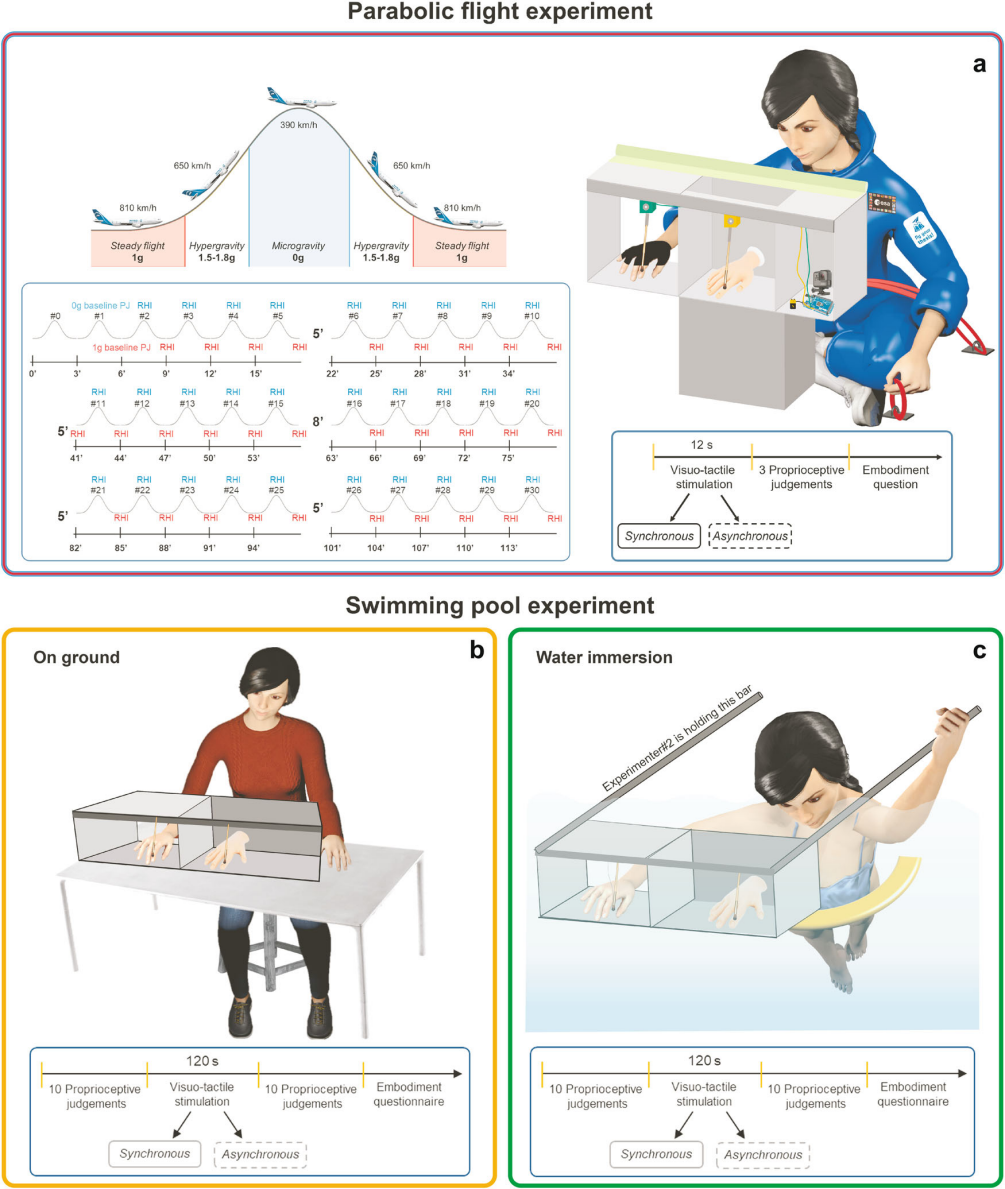
Experimental settings. Upper panel (**a**): Parabolic flight experiment. Participants underwent the RHI procedure during a parabolic flight: in normal gravity (1 g, in red) during steady horizontal flight, and in microgravity (0 g, in blue). The tactile stimulation was delivered via a fully automated system assembled ad hoc for the experiment. Lower panel (**b**, **c**): Swimming pool experiment. Participants underwent the RHI both in an ordinary laboratory (on ground condition, in orange, **b**) and in a private swimming pool (water immersion condition, in green, **c**). For graphical purposes, the coat used to cover the right shoulder and arm of participants in both experiments is not shown in the figure.

The results were the following: the proprioceptive drift (i.e., the shift of the felt position of one’s own hand toward the rubber hand), was modulated by the altered gravitational input in both experiments (see statistical details in Fig. 2b, h). In particular, during parabolic flights, a greater proprioceptive drift was found in microgravity as compared to normal gravity in both synchronous and asynchronous conditions (Fig. 2c). In the Swimming pool experiment, significant differences in the proprioceptive drift between synchronous and asynchronous conditions were found in both gravity environments (Fig. 2g, i), but, crucially, greater proprioceptive drift values were observed only after the synchronous stimulation in water immersion as compared to the ground condition (Fig. 2i). As for the embodiment questionnaire (i.e., the subjective feeling of embodying the rubber hand), a gravity-induced modulation was present in the Parabolic flight experiment only (Fig. 2e). Notably, in both experiments, the typical greater illusory experience during the synchronous than asynchronous stimulation was found irrespective of the gravity conditions (Fig. 2d, j). However, only in the Parabolic flight experiment, higher embodiment ratings were reported in microgravity relative to normal gravity in both asynchronous and synchronous stimulation, even if this latter comparison does not reach the significance level (Fig. 2l).

**Fig. 2:**
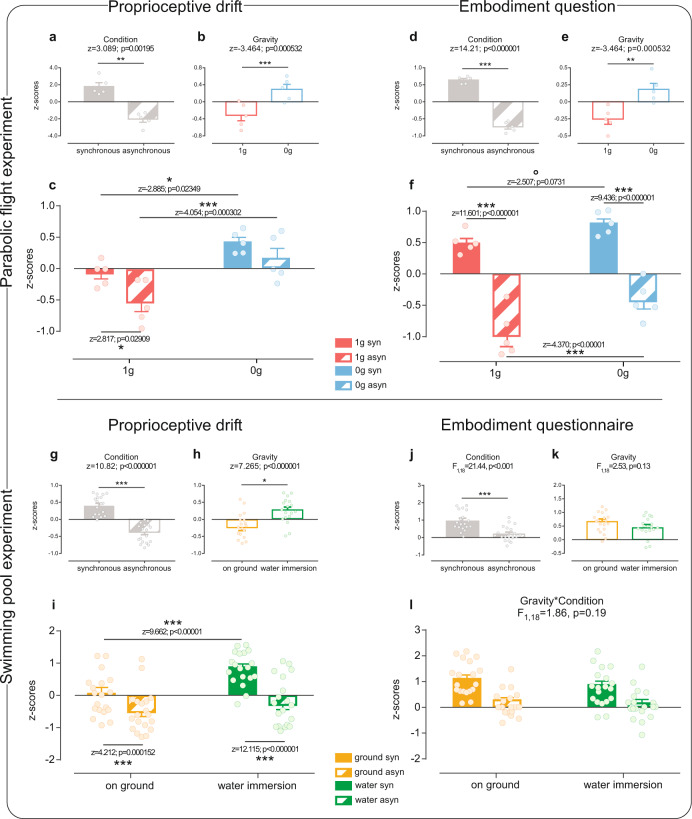
Results. Upper panel (**a**–**f**): Parabolic flight experiment. Lower panel (**g**–**l**): Swimming pool experiment. Error bars represent standard errors of the means. Dots represent single-subject values, i.e., the mean of each participant’s judgment in each condition. °tendency toward statistical significance; *p < 0.05; ***p < 0.001. Values are normalized in *z* scores.

In both experiments, altered gravity conditions (i.e., 0 g in the Parabolic flight experiment; water immersion in the Swimming pool experiment) led to a modulation of the perceived hand location, with an increased shift of the own hand position, hidden from view, toward the (visible) rubber hand. These results show that the less reliable proprioceptive inputs experienced in the gravity experimental manipulations enhance the RHI susceptibility when considering the proprioceptive drift measure after the synchronous stimulation. We propose that these results can be accounted for within the Bayesian causal inference theories of multisensory integration, which have been suggested to regulate SBO and its manipulations^9,16–18^. Individuals use probabilistic representations of their surroundings and their own body that take into account information about sensory uncertainty to infer the causal structure of sensory signals and optimally process them to create a clear perceptual distinction between the self and the nonself^16^. Therefore, when people are presented with two stimuli from different modalities, they initially infer whether these have the same origin (i.e., cause) or not, and then they combine their information according to these beliefs^19^. In the case of the RHI, the causal inference principle predicts that the rubber hand should be perceived as part of the participant’s own body if a common cause is inferred for the visual, tactile, and proprioceptive signals, meaning that when the real hand is not visible, the rubber hand might be inferred as the source of the tactile and the proprioceptive inputs^16^. In our experiments, the lack of reliable proprioceptive information (a key element for SBO to arise) caused by gravity experimental manipulations may have decreased, or even abolished, the discrepancy between sensory inputs, leading to a stronger fusion of visual and somatosensory impressions (not affected by altered gravity). As a consequence, the brain, through a probabilistic computational process, inferred that visual and tactile inputs might arise from the same source. In other words, it assumed that the rubber hand was the common cause of vision and somatosensation by dynamically taking into account all available sensory evidence given their relative reliability and prior information.

Moreover, we found additional effects only in the Parabolic flight experiment, where not only proprioception (affected during both microgravity^20^ and water immersion^21^), but also the vestibular system is perturbed^22,23^. Indeed, our vestibular system, which evolved to optimally work on ground in a 1 g environment, provides us with erroneous or disorienting information in microgravity conditions, where proprioceptive and vestibular signals^24^ become unreliable sources of information. Firstly, results show a modulation after the asynchronous (control) condition, with an increased shift toward the rubber hand and higher embodiment ratings, in microgravity than in normal gravity. This increased SBO found during the asynchronous (generally assumed to be the control) stimulation might suggest that the multisensory integration time-window is extended during microgravity. Namely, the 0 g condition, in which proprioceptive and vestibular cues are greatly attenuated, would extend the time-window in which multisensory integration occurs, inducing a stronger illusory experience even during asynchronous tactile and visual stimulations. Accordingly, it has been shown that peripheral sensory loss in vestibular-deficient patients can alter multisensory integration by reducing the ability to temporally combine sensory cues appropriately^25,26^ and making patients more susceptible to experimental manipulations of the visual inputs^27^. Secondly, results show enhanced subjective embodiment experience after the synchronous stimulation. These findings are in line with studies demonstrating a link between vestibular signals and body ownership^28,29^, and in particular, with the results of previous research proving increased subjective ratings of embodiment during the RHI after galvanic vestibular stimulation^30^ (although controversial results have been reported^31,32^). Explicit SBO is a complex and multifactorial experience and, as such, to be altered, it requires the modulation of converging multisensory information coming from our body. Accordingly, previous evidence shows that during parabolic flights, reduced intrinsic connectivity^11^ occurs in the temporoparietal junction, which is thought to contribute to the integration of vestibular, visual, and proprioceptive inputs^33^.

In summary, our results suggest that altered gravity conditions modulate the SBO, with stronger illusory embodiment of the rubber hand occurring during microgravity—where both vestibular and proprioceptive inputs are weakened—than during water immersion—where proprioception only is attenuated. Taken together, these findings contribute new evidence to our understanding of the neurocognitive mechanisms subtending our experience of the self as a unitary body. Internal brain models of multisensory integration should consider the fundamental yet neglected role of gravitational input in shaping SBO, thus increasing our understanding of the cognitive alterations experienced by astronauts during spaceflight missions.

## Methods

### Participants

Overall, 25 healthy volunteers with normal or corrected-to normal vision participated in the study (14 males; mean age: 27.3 ± 3.6; years of education: 17.2 ± 1.4). Six participants (3 males; mean age: 31.6.5 ± 3.4; years of education: 17.8 ± 0.4) took part in the Parabolic flight experiment, and 19 participants (11 males; mean age: 29.5 ± 3.45; years of education: 25.8 ± 2.3) took part in the Swimming pool experiment. Since a participant of the Parabolic flight experiment got sick during the flight, he was excluded from the analysis; therefore the sample of the Parabolic flight experiment resulted in five participants (2 males; mean age: 32 ± 3.7; years of education: 17.8 ± 0.4). All the participants of the Parabolic flight experiment had flown before on parabolic flights (i.e., they all underwent at least 31 parabolas before taking part to the present study). All participants were right-handed, as assessed with the Edinburgh Handedness Inventory^34^, naïve to the experimental procedure and gave their written informed consent to take part to the study. None of them had a history of neurological, major medical, or psychiatric disorders. The study was approved by the local ethics committee of the University of Turin (prot. n. 153210), by the ESA medical board, and by the Centre Hospitalier Universitaire de Caen, (Caen, France). All clinical investigations have been conducted according to the principles expressed in the Declaration of Helsinki.

### Reporting summary

Further information on research design is available in the Nature Research Reporting Summary linked to this article.

## Experimental procedures and set-up

### Parabolic flight experiment

#### Parabolic flight

The parabolic flights took place during the ESA parabolic flights campaign (Fly Your Thesis!2017 programme), onboard the Airbus A-310 Zero-G, in December 2017. The flights departed from Bordeaux-Mérignac airport (France) and were exploited by Novespace (www.novespace.fr). The parabolic flight campaign consisted of three different flights on 3 consecutive days. The flight consisted of three phases: a 30 s “pull-up”, during which the gravity load gradually increased to 2 g; a ~20 s “push-over” into microgravity, achieved by reducing the engine power to 60%; and a “pull-out”, during which the gravity load increased to 1.8 g. Gravity then returned to 1 g. (Fig. 1 upper panel). Every flight comprised 31 parabolas (see Supplementary Fig. 1, bottom panel), and it lasted approximately 36 h. The flight maneuver of a typical parabola is described in Supplementary Fig. 1 (upper panel; see also^35^).

#### Experimental set-up

In each flight, the experimental setting comprised one experimenter and one participant comfortably seated with crossed legs on the ground facing each other, with the experimental apparatus standing between them. Participants’ trunk midline was approximately 15 cm to the left of the RHI box center and participants were anchored to the ground via two seatbelts, one over their knees and one across their hips (Fig. 1, upper panel). The RHI box was a 65 × 42 × 22 cm polycarbonate structure standing on a 22-cm high support. The box was divided into three sections by means of two panels: from left to right, the first section (15 × 42 × 22 cm) contained the electronic devices for the tactile stimulation procedure and recording (see details below); the second section (25 × 42 × 22 cm) was open to the view; the third section (25 × 42 × 22 cm) was covered. The whole experimental set-up raised approximately 42 cm from the ground in correspondence with the participants’ chest. Participants placed their knees underneath the RHI box and slightly leaned forward while holding a strap fixed to the ground with their left hand. The right hand (i.e., the one exposed to the RHI procedure) and forearm were inserted into the right covered section of the RHI box, hidden from view, for the whole duration of the experiment. To avoid free-floating during microgravity, the right hand wore a half- glove with Velcro tape underneath, and the right index finger was fixed 15 cm to the right of the RHI box center (30 cm from the participant’s midline; Fig. 2). Participants could not see their right hand, and an additional black cloak prevented them from seeing their own shoulder. The (right) rubber hand was inserted in the central section of the box, open to the view, and it was fixed in an anatomically compatible position aligned with participants’ right shoulder so that it was perceived as an extension of the participant’s own arm. Two differently shaped rubber hands were used for females and males (Fig. 2).

The tactile stimulation was delivered via a fully automated system controlled by Arduino^®^ hardware (https://www.arduino.cc/). Two small soft paintbrushes, powered by small motors triggered by experimenters’ button presses, were stroking the upper part of the real and rubber right index fingers with a 1 Hz frequency for 12 s^10,32,36^. The tactile stimulation could be synchronous or asynchronous. During synchronous stimulation (the one expected to induce the illusion) the two brushes were touching the rubber and real finger simultaneously; during asynchronous stimulation (the one supposed not to cause the illusion) the two strokes were spatially and temporally out of phase. Synchronous and asynchronous stimulations were alternated across trials in a pseudo-random order so that the same stimulation condition was not delivered more than twice in a row. Stimulation conditions were equally distributed throughout the duration of the flight.

#### RHI measurements

To quantify the RHI effects, both subjective and objective measures were collected. As a subjective measure, we took advantage of the third question of the “Embodiment questionnaire”^6^, namely “I felt as if the rubber hand were my hand”, investigating the feeling of ownership over the fake hand. Participants were asked to evaluate the vividness of their experience of ownership over the rubber hand using a 7-point Likert scale by rating their agreement/disagreement with the item (–3 = strong disagreement; +3 = strong agreement; 0 = neither agreement nor disagreement). As an objective measure, we calculated the proprioceptive drift, i.e., the difference between the perceived position of the right index finger before and after the RHI stroking period (see details in the experimental procedures below). During the proprioceptive judgments, both the participant’s right hand and the rubber hand were kept out of view. The experimenter positioned a foldable ruler on the box along the entire edge of the RHI box on the experimenter’s side approximately 40 cm away from the participant’s chest. Participants were asked to judge the location of their index finger by verbally reporting the number on the ruler corresponding to the felt location of their right index finger. According to a pre-established sequence unknown to participants, the ruler was presented in a slightly different horizontal position on each trial, minimizing the possibility that participants used a specific number of the ruler as a landmark. Verbal answers were automatically recorded by a tie clip microphone close to the participants’ neck, connected to a continuously recording digital camera (GoPro Hero 5, https://gopro.com/).

#### Experimental procedures

Approximately 1 h before take-off, all participants (experimenters and experimental subjects) were administered a subcutaneous injection of scopolamine by the campaign medical doctor, as it is routinely the case before parabolic flights^37^ (see details about medical administration below). It is important to highlight that scopolamine was equally effective for the whole duration of the parabolic flight, thus affecting all experimental conditions similarly. Therefore, the drug injection alone cannot be considered responsible for the selective modulation of any experimental condition. As described above, each of the 31 parabolas consisted of three phases: 20 s of hypergravity, 22 s of microgravity, 22 s of hypergravity again. Onboard, during the first parabola, the experimenter and the experimental subjects stayed still to adapt to the feeling of gravity transitions. In between parabolas, there were ~90 s of break, with stable horizontal flight and normal gravity. The second parabola was dedicated to baseline proprioceptive judgments, to obtain baseline data regarding participants’ perceived position of their right index finger without any experimental manipulation. Five proprioceptive judgments were collected both during the microgravity phase (i.e., 0 g) and immediately after, during the break (i.e., steady flight, 1 g). The whole proprioceptive judgment collection phase lasted approximately 10 s. For the remaining 28 parabolas (i.e., 0 g condition) and 28 breaks in between them (i.e., 1 g condition), the experimental procedures were identical, as follows. The last parabola was used as a backup if something went wrong in the previous procedures. After 12 s^38^ of tactile stimulation (synchronous or asynchronous, depending on the condition), as soon as the stimulation was over, the experimenter pulled a rolling curtain over the top of the RHI box to cover homogeneously both the rubber hand and the entire surface of the box in a way not to provide any spatial reference frame to the participant. Then, the experimenter placed the foldable ruler on the box to collect the participant’s proprioceptive judgment, immediately followed by the question regarding the feeling of body ownership. Once every five parabolas, a longer break allowed participants to stand up and stretch their legs (see Fig. 1, upper panel). A total of 56 proprioceptive judgments and 56 body ownership ratings per subject were collected: 14 after the synchronous stimulation in 0 g, 14 after the asynchronous stimulation in 0 g, 14 after the synchronous stimulation in 1 g, and 14 after the asynchronous stimulation in 1 g. The beginning of each gravity condition (1 and 0 g) was signaled to the experimenter both by an accelerometer placed over the experimental box and by an acoustic signal triggered by the airplane’s pilot.

Following the suggestion of an anonymous reviewer, we carried out an additional control experiment to control if, in the 0 g condition of the Parabolic flight experiment, a Jendrassik effect (i.e., a method for enhancing sluggish tendon-tap jerks evoked at medical examination) influenced body muscle tonus broadly during the RHI procedure. See details in Supplementary Information.

#### Acceleration data and flight maneuvers

At the beginning and end of each parabola, when the engines were on with the airplane nose high and low at 45° respectively, two hypergravity phases lasting about 10 s occurred. During hypergravity, subjects remained still with their eyes closed because of the high probability of motion sickness. As said above, the experiments were performed only during the 0 and 1 g phases. In between different gravity phases, there were short transition phases. An acoustic signal signaled to the experimenter the end of each transition phase and the beginning of a stable gravity condition. The 0 g phase, during free fall, is characterized by a stable gravity condition with g force oscillating between 0.03 and −0.03 g. The periods of weightlessness last approximately 22 s. The complete acceleration data of a whole flight have been deposited in Mendeley (10.17632/m37ktzyfn6.1), and the flight maneuver of a typical parabola is presented in Supplementary Fig. 1 (upper panel). It is important to point out that normal gravity condition was less stable due to normal turbulences during horizontal flight. However, as it can be appreciated in Supplementary Fig. 1 (bottom panel), g levels rarely exceeded 1.2 and 0.8 g, while normal oscillations were within the range of ±0.05 g.

#### Anti-nausea medication

Coherently with previous neuroscientific studies^35,39^, an anti-nausea medication (i.e., scopolamine) was administered 1.5 h before flight to prevent motion sickness caused by hypergravity. Doses corresponded to 0.125 mg for females and 0.175 mg for males. Such dosage is lower than commercial treatments prescribed for motion sickness and chemotherapy-induced nausea, which generally have no side effects. Commonly reported side effects (generally for higher dosages) of scopolamine include drowsiness and xerostomia; less common side effects include enlarged pupils, confusion, increased sensitivity of the eyes to light. None of the participants in our study reported any side effects.

### Swimming pool experiment

#### Experimental set-up

The experiment was carried out in two different environments: in a standard laboratory (i.e., on ground condition) and in a swimming pool ~1.5 m deep (i.e., experimental condition). In both sessions, the RHI procedure was performed employing a box (86 × 35 × 22 cm) made with alveolar polypropylene, a material with good floating and water-repelling properties. The box was divided into two equal halves (43 × 35 × 22 cm) by a panel. One of the two halves was open to the view to allow viewing the rubber hand, while the other half remained covered to prevent viewing of the subject’s right hand. Two apertures on both horizontal sides of the box hosted the participant’s arm and the rubber hand. A black waterproof towel covered the subject’s shoulders and the proximal end of both the subject’s right hand and the rubber hand so that the rubber hand appeared as an extension of the participant’s arm. The box was placed in front of the subject’s chest (distant about 15 cm) so that the rubber hand, placed in the half of the box open to the view, was aligned with the participant’s right shoulder. In the swimming pool session, participants effortlessly float with the aim of a bar float placed under the chest. Therefore, the experimental subject was facing the RHI box, floating without touching the pool bottom with his/her feet. Furthermore, participants hold a metal bar fixed to the upper part of the RHI box with their left hand to maintain balance, while the right hand (i.e., the one undergoing the RHI procedure) was placed inside the box (Fig. 1, lower panel). To avoid movements caused by the water, the real (right) and the rubber hand were both sustained by employing a transparent fishing wire fixed to the box.

The experimenter, facing the subject, administered the tactile stimulation to the participant’s right index finger and to the rubber hand’s index finger with two paintbrushes. The stimulation could be synchronous or asynchronous, depending on the experimental condition.

#### RHI measurements

To quantify the RHI effects, both subjective and objective measures were collected. As subjective measure, we employed the “Embodiment questionnaire”^6^ to investigate the feeling of ownership over the rubber hand (see details in Table 1). The questionnaire contains three target and three control statements^6^. Participants were asked to evaluate the vividness of their experience of ownership over the rubber hand using a 7-point Likert scale by rating their agreement/disagreement with each item (–3 = strong disagreement; +3 = strong agreement; 0 = neither agreement nor disagreement). It is worth noticing that, because each participant underwent four identical sessions, the items of the two questionnaires were presented in random order.

**Table 1:** Embodiment questionnaire (Botvinick and Cohen^6^).

1. It seemed as though the touch I felt was caused by the paintbrush touching the rubber hand
2. It seemed as if I were sensing the touch of the paintbrush in the location where I saw the rubber hand touched
3. I felt as if the rubber hand were my hand
4. It felt as if my hand were drifting toward the left/right (toward the rubber hand)
5. It seemed as if the touch I was feeling came from somewhere between my own hand and the rubber hand
6. It felt as if my hand were turning “rubbery”

The questionnaire consists of six selected statements (1–3 target questions, 4–6 control questions) from a previous study (Botvinick and Cohen^6^). Participants were asked to evaluate the vividness of their experience of ownership over the rubber hand using a 7-point Likert scale, by rating their agreement/disagreement with each item (–3 = strong disagreement; +3 = strong agreement; 0 = neither agreement nor disagreement).

As an objective measure, we used the proprioceptive drift, computed as the difference between the perceived position of the right index finger before and after the RHI stroking period (see details in the experimental procedures below). During the proprioceptive judgments, both the participant’s right hand and the rubber hand were concealed from view. The experimenter positioned a foldable ruler on the box along the edge of the RHI box facing him. Participants were asked to judge the location of their index finger by verbally reporting the number on the ruler corresponding to the felt location of their index finger. The ruler was presented with a randomly displaced onset to ensure that participants judged finger position anew on each trial and could not spatially anchor to previous responses.

#### Experimental procedures

Each participant underwent four RHI conditions in two different sessions: one in a standard laboratory (i.e., on ground condition) and one in a private swimming pool (i.e., water immersion condition). In each session, participants underwent the RHI both with synchronous and asynchronous stroking, with 1 h minimum delay between sessions. The order of the sessions and conditions was counterbalanced among subjects. After familiarization with the experimental procedures, the experimenter covered both the real and the rubber hand. The subject started to report verbally (i.e., ten trials) the perceived position of the own index finger (pre-proprioceptive judgment) according to the numbers of the ruler randomly positioned by the experimenter. Then, the experimenter showed the rubber hand and started stroking (synchronously or asynchronously, depending on the condition) the participants’ index finger and the rubber hand index finger for 120 s. After the tactile stimulation, participants were asked again to report the perceived horizontal position (i.e., ten trials; post-proprioceptive judgment) of their index finger and filled out the questionnaire assessing the subjective experience of the illusion.

## Data analysis

### Parabolic flight experiment

Single subjects’ answers (proprioceptive judgments, questionnaire ratings) were extracted from audio recordings after each flight. Due to the rough experimental conditions, some answers went missing because of technical issues (on average 4.3 trials over a total of 56 measurements per participant; the number of the valid trials was comparable among experimental conditions). Outliers (judgments deviating more than 2.5 standard deviations from single subjects means) were removed and excluded from subsequent analyses (one judgment per participant was excluded on average in each condition). The proprioceptive drift was calculated separately for 1 and 0 g conditions. In particular, concerning 1 g condition, the proprioceptive drift was computed as the difference between the mean of the proprioceptive judgments collected immediately after the second parabola in steady flight condition (i.e., baseline proprioceptive judgments in 1 g) and each of the proprioceptive judgments collected after each RHI procedure (i.e., the indicated location of the participant index finger after the stroking period); concerning 0 g condition, the proprioceptive drift was computed as the difference between the mean of the proprioceptive judgments collected during the second parabola in microgravity condition (i.e., baseline proprioceptive judgments in 0 g) and each of the proprioceptive judgments collected after each RHI procedure during microgravity (i.e., the indicated location of the participant index finger the stroking period). All the observations were normalized in *z* scores separately for the proprioceptive drift and the body ownership question, calculated within-subjects across conditions (i.e., 1 g synchronous, 1 g asynchronous, 0 g synchronous, 0 g asynchronous), and entered in a linear mixed model (LMM) analysis. Hence, we ran separate LMM analyses—one for proprioceptive drift and one for the body ownership question^40–42^—in R (version 4.0.0, https://www.r-project.org/), using the lme4 package^43^. In both LMM models, we included the proprioceptive drift and the body ownership question as dependent variables, and we parameterized them into the combined variable Gravity (1 g; 0 g) and Condition (synchronous; asynchronous), resulting in the following conditions: 1 g synchronous; 1 g asynchronous; 0 g synchronous; 0 g asynchronous. For the proprioceptive drift and for the body ownership question, we separately investigated the main effects of Gravity (1 g vs 0 g, irrespective of the condition) and Condition (synchronous vs asynchronous, irrespective of the gravity), and then the specific effects within the Gravity Condition parameterization. Hence, we ran, between conditions, simultaneous tests for general linear hypotheses with multiple comparisons of the means by employing Tukey contrasts (Bonferroni corrected). Participants’ age and gender were added as fixed effects and subjects’ ID as a random effect. We used LMMs because these analyses allowed us to account for variability due to trial type in our model while simultaneously accounting for the fact that trials were nested within-subjects, and that multiple responses from the same person are more similar than responses from other people. Accounting for both trial type and subject-level variance in objective and subjective judgments was expected to reduce the error in our models and increase the statistical power despite the small sample size. In Fig. 2a–f, we reported *z* and *p* values for each analysis.

### Swimming pool experiment

For each subject, the mean of the pre-proprioceptive judgments was subtracted from the mean of the post-proprioceptive judgments and referred to as proprioceptive drift. Concerning the Embodiment questionnaire, we separately averaged ratings of “target” questions (1–3 statements that reveal the presence of the illusion) and ratings of the “control” statements (4–6 statements used as control, see Table 1)^44^. To compute the main effect of the subjective experience of owning the fake hand, we then subtracted the average of control ratings from the average of target ones^45^. Data were normalized in *z* scores separately for the proprioceptive drift and the body ownership questionnaire, calculated within-subjects across conditions (i.e., on ground synchronous, on ground asynchronous, water immersion synchronous, water immersion asynchronous).

We partially adopted the same analyses employed in the Parabolic flight experiment, and we ran LMM analysis with all the judgments about the hand location (i.e., proprioceptive drift) as dependent variable in R (version 4.0.0, https://www.r-project.org/), using the lme4 package^43^. We parameterized the variable into the combined variable Gravity (on ground; water immersion) and Condition (synchronous; asynchronous), resulting in the following conditions: on ground synchronous; on ground asynchronous; water immersion synchronous; water immersion asynchronous. Similarly to the parabolic flight experiment, we investigated the main effects of Gravity (on ground; water immersion, irrespective of the condition) and Condition (synchronous vs asynchronous, irrespective of the gravity), and then the specific effects within the Gravity Condition parameterization. In Fig. 2g–i, we reported *z* and *p* values for each analysis. Concerning the embodiment questionnaire, since it requires only one rating for each question, the random factor Subject was not employed, and therefore we employed a generalized linear model, i.e., a 2 × 2 repeated measures ANOVA with Gravity (two levels: on ground; water immersion) and Condition (two levels: synchronous; asynchronous) as within-subjects factors, performed with Statistica software 8.0 (StatSoft, Inc., Tulsa, OK, USA). According to Shapiro–Wilk test, residuals were normally distributed (*p* > 0.05). Post hoc comparisons were performed employing Newman–Keuls’s test. Figure 2j–l reports *F* and *p* values.

## Supplementary information

Supplementary Information

Reporting Summary

## Data Availability

Anonymized data have been deposited in Mendeley (10.17632/m37ktzyfn6.1).
