# Supplementary material for: The rubber hand illusion in microgravity and water immersion
Source: NPJ Microgravity. 2022 May 6;8:15. doi: 10.1038/s41526-022-00198-4 (PMC9076892; doi:10.1038/s41526-022-00198-4)
Supplement: Supplementary file 1 — Supplementary Information [file 41526_2022_198_MOESM1_ESM.pdf]

Supplementary Information

Acceleration data figure

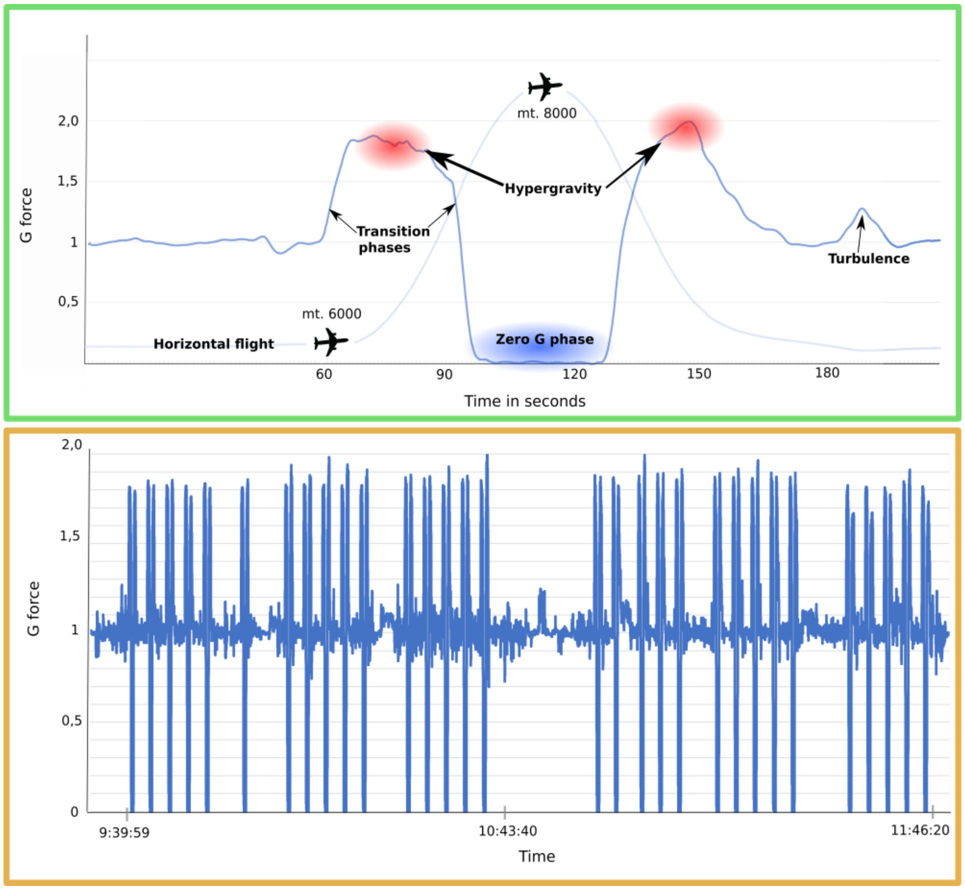

Supplementary Figure 1. The *Upper Panel* shows the acceleration (g) and the trajectory of a typical parabola (data were chosen randomly from day 1 flight), with Hypergravity, Zero-G (0g), 1g, and transition phases. A full parabola (between two steady horizontal flight phases) lasted about 70 seconds. The *Bottom Panel* shows acceleration (g) data from a whole flight performed on day 1.

Control experiment

A control experiment was carried out to control if, in the 0g condition of the Parabolic flight experiment, a Jendrassik effect (i.e., a method for enhancing sluggish tendon-tap jerks evoked at medical examination) influenced body muscle tonus broadly during the RHI procedure. To investigate this aspect, in this control experiment, participants underwent the RHI while holding a strap belt, fixed to the floor, in their left (not-illuded) hand in two conditions: in the No force condition, participants naturally held the strap belt without putting any force; in the Force condition, they were asked to held the strap putting more power and keeping tight the muscles. This latter condition was employed to elicit the Jendrassik effect.

Methods

Participants

Sixteen (5 men, mean age – SD: years = 24.8 – 2.6; years of education = 17.8 – 1.4) healthy volunteers were recruited for the control experiment. All participants were right-handed, as assessed with the Edinburgh Handedness Inventory (Oldfield, 1971), naive to the experimental procedure, and before taking part in the study, they gave written informed consent. None of them had a history of neurological, major medical, or psychiatric disorders. The experimental procedure was approved by the local Ethics Committee of the University of Turin (prot. n. 133278, 07/03/19).

Experimental set-up and procedures

The very same set-up employed in the Parabolic flight experiment was used in the present experiment aiming at controlling a possible elicitation of a Jendrassik effect, that would have potentially influenced body muscle tonus broadly during 0g in the Parabolic flight experiment. In the present control experiment, the same experimental apparatus and automated tactile stimulation were used. The experimental procedures mirrored the Parabolic flight experiment's ones, with the exception that the control experiment was carried out in an ordinary laboratory (with normal gravity). The first trial was dedicated to baseline proprioceptive judgments, to obtain baseline data regarding participants' perceived position of their right index finger without any experimental manipulation (i.e., No force condition). Five proprioceptive judgments were collected. The second trial was dedicated to baseline proprioceptive judgments in the Force condition, the one employed to elicit a Jendrassik effect. To do so, participants were asked to hold a strap with the left hand, and to pull it with their maximum force. Importantly, participants hold the same strap in the No force condition, but without using any force (i.e., the strap was passively held in the hand). In the remaining 56 trials (i.e., 28 trials of No force and 28 trials of Force condition), the RHI was induced, and the experimental procedures were identical to the Parabolic flight experiment' ones (see main text for details on how the RHI was performed). After each trial of RHI administration, we collected objective and subjective RHI measurements. In other words, we collected a total of 56 proprioceptive judgments and 56 body ownership ratings per subject: 14 after the synchronous stimulation in No force condition, 14 after the asynchronous stimulation in No force condition, 14 after the synchronous stimulation in Force condition, and 14 after the asynchronous stimulation in Force condition. Electromyographic (EMG) activity was recorded during each trial to assure that participants passively (in the No force condition) or actively (in the Force condition) held the strap.

EMG recording

EMG activity was recorded from the flexor digitorum communis of participants' right arm by pairs of Ag–AgCl surface pre-gelled electrodes (24mm diameter), following standard skin preparation. The electrodes were connected to a Biopac MP-150 electromyograph (Biopac Systems Inc., Santa Barbara, CA). The EMG signal was acquired at 10 kHz sampling rate, amplified, filtered with a band-pass (10–500 Hz) and a notch (50 Hz) filter and stored on a PC for offline analysis. Each recording epoch lasted about 18 seconds: the automated system device for RHI stroking triggered the EMG at the beginning of the (12 seconds) tactile stimulation, and an experimenter manually ended the EMG epoch recording after the participant's rating about body ownership.

Data Analyses

As for the Parabolic flight experiment, single subjects' answers (proprioceptive judgments, questionnaire ratings) were analyzed. The proprioceptive drift was calculated separately for No force and Force conditions as the difference between the mean of the proprioceptive judgments collected in the first and in the second trials, respectively, and each of the proprioceptive judgments collected after each RHI procedure. All the observations were normalized in z-scores separately for the proprioceptive drift and the body ownership question, calculated within-subjects across conditions (i.e., synchronous No force, asynchronous No force, synchronous Force, asynchronous Force), and entered in a Linear Mixed Model (LMM) analysis. Hence, we ran separate LMM analyses – one for proprioceptive drift and one for the body ownership question – in R (version 4.0.0, https://www.r-project.org/), using the lme4 package28. In both LMM models, we included the proprioceptive drift and the body ownership question as dependent variables, and we parameterized them into the combined variable Strength (No force; Force) and Condition (synchronous; asynchronous), resulting in the following conditions: synchronous No force, asynchronous No force, synchronous Force, asynchronous Force. For the proprioceptive drift and for the body ownership question, we separately investigated the main effects of Strength (No force vs Force, irrespective of the condition) and Condition (synchronous vs asynchronous, irrespective of the strength), and then the specific effects within the Strength Condition parameterization. Hence, we ran, between conditions, simultaneous tests for general linear hypotheses with multiple comparisons of the means by employing Tukey contrasts (Bonferroni corrected). Participants' age and gender were added as fixed effects and subjects' ID as a random effect. We used LMMs to mirror the Parabolic flight's experiment analyses. The EMG activity of the flexor digitorum communis was recorded in each subject and represented as a root- mean-square (RMS) value. Again, a LMM model was performed including the RMS as the dependent variable. Since the aim of this analysis was to ensure that participants passively (No Force condition) or actively (Force condition) held the strap belt, we compared the EMG activity acquired in these two conditions, regardless of the RHI tactile stimulation (synchronous vs asynchronous).

Results

Both in the objective (i.e., proprioceptive drift) and subjective (i.e., embodiment question) measurements, a significant effect of Condition was found (proprioceptive drift: $z=24.28$; $p< 0.0000001$; embodiment question: $z= 39.59$; p\textless 0.0000001), with, as expected, higher values after the synchronous than after the asynchronous stimulation. Therefore, the classical RHI effect emerged. Importantly, we did not find a Strength effect (proprioceptive drift: $p=0.717$; embodiment question: $p=0.428$) and effect in Strength Condition parameterization (proprioceptive drift: No force syn vs Force syn: $p=1$; No force asyn vs Force asyn: $p=1$; embodiment question: No force syn vs Force syn: $p=1$; No force asyn vs Force asyn: $p=1$;), suggesting that the recruitment of body muscles in the Force condition did not affect the classical RHI experience (as in the No Force condition). Importantly, the EMG results confirmed that participants recruited body muscles in the left (not illuded) hand only in the Force condition (i.e., when they actively held the strap belt, as to elicit a Jendrassik effect; No Force vs Force: <0.0001). Altogether the results of this control experiment suggest that even if, in the 0g condition of the Parabolic flight experiment, a Jendrassik effect had been elicited, it did not influence body muscle tonus broadly during the RHI procedure. Therefore, the modulation of RHI measurements obtained in the microgravity condition of the Parabolic flight experiment cannot be ascribed to a pure "muscular" effect.

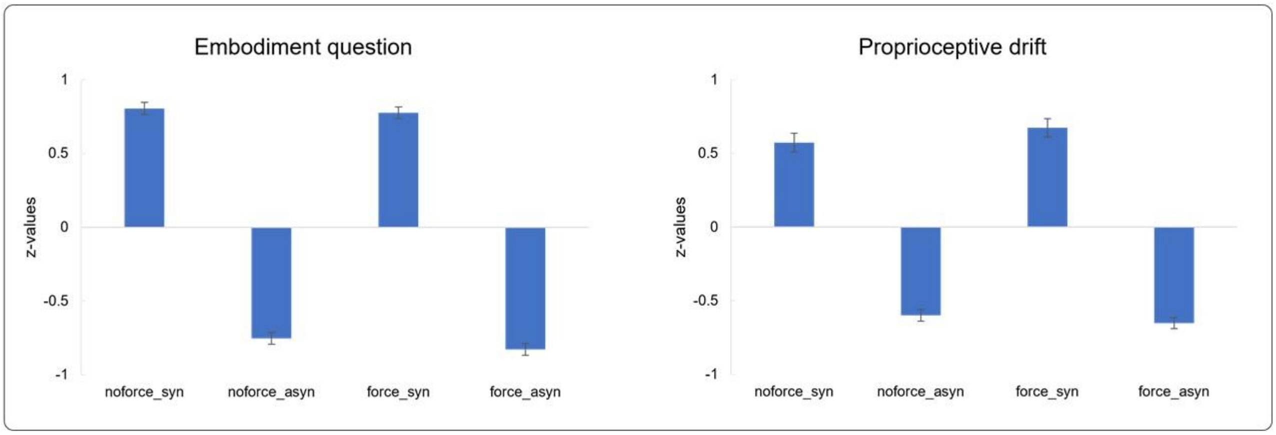

Supplementary Figure 2. The *Left Panel* shows the objective proprioceptive drift average z-normalized data. Th*e Right Panel* shows subjective embodiment data.
