# Supplementary material for: The rubber hand illusion in microgravity and water immersion
Source: NPJ Microgravity. 2022 May 6;8:15. doi: 10.1038/s41526-022-00198-4 (PMC9076892; doi:10.1038/s41526-022-00198-4)
Supplement: Supplementary file 2 — Reporting Summary [file 41526_2022_198_MOESM2_ESM.pdf]

nature portfolio

Corresponding author(s): Pietro Sarasso, NPJMGRAV-00671R

Last updated by author(s): 08/03/2022



# Reporting Summary

Nature Portfolio wishes to improve the reproducibility of the work that we publish. This form provides structure for consistency and transparency in reporting. For further information on Nature Portfolio policies, see our Editorial Policies and the Editorial Policy Checklist.

## Statistics

For all statistical analyses, confirm that the following items are present in the figure legend, table legend, main text, or Methods section.

| n/a | Confirmed | |
|---|---|---|
| ☐ | ☒ | The exact sample size ($n$) for each experimental group/condition, given as a discrete number and unit of measurement |
| ☐ | ☒ | A statement on whether measurements were taken from distinct samples or whether the same sample was measured repeatedly |
| ☐ | ☒ | The statistical test(s) used AND whether they are one- or two-sided<br>*Only common tests should be described solely by name; describe more complex techniques in the Methods section.* |
| ☐ | ☒ | A description of all covariates tested |
| ☐ | ☒ | A description of any assumptions or corrections, such as tests of normality and adjustment for multiple comparisons |
| ☐ | ☒ | A full description of the statistical parameters including central tendency (e.g. means) or other basic estimates (e.g. regression coefficient) AND variation (e.g. standard deviation) or associated estimates of uncertainty (e.g. confidence intervals) |
| ☐ | ☒ | For null hypothesis testing, the test statistic (e.g. $F$, $t$, $r$) with confidence intervals, effect sizes, degrees of freedom and $P$ value noted<br>*Give P values as exact values whenever suitable.* |
| ☒ | ☐ | For Bayesian analysis, information on the choice of priors and Markov chain Monte Carlo settings |
| ☒ | ☐ | For hierarchical and complex designs, identification of the appropriate level for tests and full reporting of outcomes |
| ☒ | ☐ | Estimates of effect sizes (e.g. Cohen's $d$, Pearson's $r$), indicating how they were calculated |

*Our web collection on statistics for biologists contains articles on many of the points above.*

## Software and code

Policy information about availability of computer code

| | |
|---|---|
| Data collection | Arduino® hardware (https://www.arduino.cc/); GoPro Hero 5, https://gopro.com/). |
| Data analysis | R (version 4.0.0, https://www.r-project.org/) lme4 package; Statistica software 8.0 (StatSoft, Inc., Tulsa, OK, USA) |

For manuscripts utilizing custom algorithms or software that are central to the research but not yet described in published literature, software must be made available to editors and reviewers. We strongly encourage code deposition in a community repository (e.g. GitHub). See the Nature Portfolio guidelines for submitting code & software for further information.

## Data

Policy information about availability of data

All manuscripts must include a data availability statement. This statement should provide the following information, where applicable:

- Accession codes, unique identifiers, or web links for publicly available datasets
- A description of any restrictions on data availability
- For clinical datasets or third party data, please ensure that the statement adheres to our policy

http://dx.doi.org/10.17632/m37ktzyfn6.1

# Field-specific reporting

Please select the one below that is the best fit for your research. If you are not sure, read the appropriate sections before making your selection.

☒ Life sciences ☐ Behavioural & social sciences ☐ Ecological, evolutionary & environmental sciences

For a reference copy of the document with all sections, see nature.com/documents/nr-reporting-summary-flat.pdf

# Life sciences study design

All studies must disclose on these points even when the disclosure is negative.

**Sample size**

In the Parabolic flight experiment, the sample was of 6 participants since the Parabolic Flight Campaign allowed to recruite this number of experimental subjects (2 participants per flight, 3 flights in 3 days).
In the Swimming pool experiment, 19 participants were recruited according to our previuos studies on Rubber Hand Illusion, in which the sample was of at least 14 subjects:
- Fossataro et al., 2018, EJN: 16 participants
- Rossi Sebastiano et al., 2021, Psychological Research: 18 participants
- Pyasik et al., 2021, NeuroImage: 14 participants
- Dell'Anna et al., 2018, Neuropsychologia: 19 participants

**Data exclusions**

Since a participant of the Parabolic flight experiment got sick during the flight, he was excluded from the analysis; therefore the sample of the Parabolic flight experiment resulted in five participants (2 males; mean age: 32±3.7; years of education: 17.8±0.4).

Due to the rough experimental conditions, of the Parabolic Fligh experiment some answers went missing because of technical issues (on average 4.3 trials over a total of 56 measurements per participant; the number of the valid trials was comparable among experimental conditions). Outliers (judgments deviating more than 2.5 standard deviations from single subjects means) were removed and excluded from subsequent analyses (one judgment per participant was excluded on average).

**Replication**

In both experiments, proprioceptive drift and subjective emobodiment ratings were tested as measures of rubber hand illusion. The data obtained in the baseline conditions (i.e., in 1g in the Parabolic Flight experiment, and on ground in the Swimming pool experiment) replicate the findings of the classical literature on Rubber Hand Illusion effects.

**Randomization**

This study comprised two experiments in two different context (and nations: one in France and one in Italy), therefore subjects of each experiment were recruited in different times and places. Moreover, in each experiment, each experimental subected underwent the same experimental condition. In the Parabolic flight, the experimental conditions were alternated across trials in a pseudo-random order so that the same stimulation condition was not delivered more than twice in a row. In the Swimming pool experiment, the order of the experimental (water immersion, on ground) sessions and conditions (synchronous, asynchronous) was counterbalanced among subjects.

**Blinding**

In this study the contribution of the experimenter was extremely important, since he/she is striclty involved in the experimental procedures (e.g., he/she had to measure the participant's responses, to chose the stimulation condition, to register the behavioral responses). For this reason, the experimenter was not blind.

# Reporting for specific materials, systems and methods

We require information from authors about some types of materials, experimental systems and methods used in many studies. Here, indicate whether each material, system or method listed is relevant to your study. If you are not sure if a list item applies to your research, read the appropriate section before selecting a response.

## Materials & experimental systems

| n/a | Involved in the study | |
|---|---|---|
| ☒ | ☐ | Antibodies |
| ☒ | ☐ | Eukaryotic cell lines |
| ☒ | ☐ | Palaeontology and archaeology |
| ☒ | ☐ | Animals and other organisms |
| ☐ | ☒ | Human research participants |
| ☒ | ☐ | Clinical data |
| ☒ | ☐ | Dual use research of concern |

## Methods

| n/a | Involved in the study | |
|---|---|---|
| ☒ | ☐ | ChIP-seq |
| ☒ | ☐ | Flow cytometry |
| ☒ | ☐ | MRI-based neuroimaging |

## Human research participants

Policy information about studies involving human research participants

**Population characteristics**

Overall, twenty-five healthy volunteers with normal or corrected-to normal vision participated in the study (14 males; mean age: 27.3±3.6; years of education: 17.2±1.4). Six participants (3 males; mean age: 31.6.5±3.4; years of education: 17.8±0.4)

| | |
|---|---|
| | took part in Parabolic flight experiment, and nineteen participants (11 males; mean age: 29.5±3.45; years of education: 25.8 ±2.3) took part in the Swimming pool experiment. Since a participant of the Parabolic flight experiment got sick during the flight, he was excluded from the analysis; therefore the sample of the Parabolic flight experiment resulted in five participants (2 males; mean age: 32±3.7; years of education: 17.8±0.4). |
| Recruitment | In the Parabolic flight experiment, the participants were recruited for having flown before on parabolic flights (i.e., they all underwent at least 31 parabolas before taking part to the present study).<br>In the Swimming pool experiment, participants were recruited from the database of experimentas subjects of the Department of Psychology from the University of Turin. |
| Ethics oversight | The study was approved by the local ethics committee of the University of Turin (prot. n. 153210), by the European Space Agency (ESA) medical board, and by the Centre Hospitalier Universitaire de Caen, (Caen, France). |

Note that full information on the approval of the study protocol must also be provided in the manuscript.
